# Development and Psychometric Test of the Salutogenic Survey on Sustainable Working Life for Nurses: Identifying Resistance Resources against Stress

**DOI:** 10.3390/ijerph21020198

**Published:** 2024-02-08

**Authors:** Monica Eriksson, Elias Johannesson, Nóra Kerekes, Maria Emilsson, Sandra Pennbrant, Håkan Nunstedt

**Affiliations:** 1 Department of Health Sciences, University West, SE-46186 Trollhättan, Sweden; monica.eriksson@hv.se (M.E.); nora.kerekes@hv.se (N.K.); maria.emilsson@hv.se (M.E.); sandra.pennbrant@hv.se (S.P.); 2 Department of Social and Behavioural Studies, University West, SE-46186 Trollhättan, Sweden; elias.johannesson@hv.se; 3 Region Västra Götaland, Intensive Care Unit, NU Hospital Group, SE-46173 Trollhättan, Sweden

**Keywords:** instrument development, Confirmatory Factor Analysis (CFA), salutogenesis, the Salutogenic Survey on Sustainable Working life for nurses (SalWork-N), generalised and specific resistance resources, specific enhancing resources

## Abstract

Extensive research shows nurses’ work environment to be particularly stressful. This study develops, explores, and psychometrically tests a new profession-specific questionnaire identifying generalised and specific resistance resources, that make it possible to measure resources to manage work-related stress. An exploratory study design was employed. The questionnaire development was inspired by the MEASURE approach and the salutogenic theory of health. Building on the results from a literature review of nursing research and salutogenesis, supplemented by twelve interviews with hospital nurses, an item pool was generated. The first version was pilot-tested in a group of nurses who were studying to become specialist nurses. The second version of the questionnaire was psychometrically tested on a sample of registered nurses in close patient care (n = 475), analysed using confirmatory factor analysis to test seven predefined domains of the questionnaire. The analysis revealed a first order seven-domain model of 21 items: job satisfaction, professional role, work motivation, commitment, belonging in the workplace, factors and conditions for remaining in the profession, and workload. The structure of the questionnaire indicates its usefulness in clinical practice for measuring resistance resources.

## 1. Introduction

Nurses play a pivotal role in the healthcare sector. A sustainable working life is a prerequisite for all nursing professionals, to maintain health and to prevent them leaving the profession and the workplace. According to Eurofound [1], sustainable work means that working and living conditions are such that they support people in engaging and remaining in work throughout an extended working life. From a salutogenic perspective, workplace health can be defined as “the ability of the workforce to participate and be productive in a sustainable and meaningful way” [2]. A salutogenic organisation provides personal, social, and environmental resources that offer coherent working experiences and sustainable organisational outcomes. It promotes the development of the capacities of employees and employers to use these resources [2], p. 77. In a nursing context the concept of sustainability is defined based on attributes such as ecology, the environment, the future, globalism, holism, and maintenance [3]. Extensive research shows that nurses experience their work situation as demanding, characterised by a high level of work-related risk factors for disease such as high workload, depression, burnout, and pandemics [4,5,6]. At the same time the shortage of nurses in Europe is so serious that it has been described as “a ticking time bomb” [7], especially in the case of specialised nurses [8], which is furthermore aggravated by the trend of nurses’ motivation to leave the profession and workplace [9,10,11,12,13,14,15]. It is therefore essential to not only avoid burnout [13,16], depression [17], and mental health problems among nurses [18], but also identify resistance resources against stress [19,20], enhancing resources for health [21], work-related “push and pull factors” [11], and even factors that make nurses thrive professionally [22,23], enabling nurses to remain in the profession and workplace.

This makes us focus on the resource-oriented instead of a risk-oriented perspective on the work environment and health, i.e., the salutogenic perspective [19,20]. Previous research shows that the salutogenic core concept sense of coherence (SOC) seems to have a moderating as well as a mediating role in buffering stress among nurses [24,25,26]. More than 30 years have passed since Antonovsky [19] developed the first questionnaire for measuring the sense of coherence (SOC), entitled The Orientation to Life Questionnaire. Over the years, various modified forms of the SOC questionnaire have been developed, with the same questions as Antonovsky but with different response alternatives and different numbers of items included in the studies. As far as we know there are no other instruments based on the salutogenic theory and specifically developed for hospital nurses. In an ever-changing world, with a shortage of nurses together with an increasing intention to leave the profession and the workplace, it becomes particularly challenging for all societies, healthcare systems, and nursing practice. Keeping these facts in mind, it is therefore important to identify the GRRs and SRRs relevant to today’s society and, particularly to the nursing context. Therefore, to fill this knowledge gap, the Salutogenic Survey on Sustainable Working Life for Nurses (SalWork-N) was developed and is presented here. The aim of the study was twofold: (1) to develop a new profession-specific questionnaire identifying resistance resources that may enhance nurses to manage stress at work; and (2) to psychometrically test the new questionnaire, entitled the Salutogenic Survey on Sustainable Working Life for Nurses (SalWork-N).

### The Salutogenic Framework

In 1987, Antonovsky [19] introduced the Orientation to Life Questionnaire, a sense of coherence scale, describing three central dimensions to manage stress, comprehensibility, manageability, and meaningfulness. In the first two decades, the focus was on validating the SOC questionnaire in different countries, cultures, and contexts, and on different populations [20]. During this time, salutogenesis was regarded the same as the SOC. However, this view has more recently been broadened. Salutogenesis is now seen as a theory, a health model, and an orientation to life [20], not only the measurement of SOC. Salutogenesis is an umbrella concept covering many theories of salutogenic and health promoting factors applicable not only at an individual level, but at group and organisational levels [20]. Along with the core concept of the SOC, there are two other central concepts to keep in mind: generalised (GRRs) and specific (SRRs) resistance resources against stress [19,20]. The function of these resistance resources is that they create conditions for people and groups (here, nursing teams) to develop strong SOC. Extensive research on the SOC gives evidence for that a strong SOC mediates as well as moderates stress [20]. In the 21st century, more attention is being paid to the relationship between the SOC and nurses’ work-related patterns of behaviour [26]. Burnout was related to a low SOC, meaning that the ability to manage stress was impaired. Work-related patterns of behaviour among Polish nurses showed that a strong SOC was associated with healthy functioning [27]. More recent, research focused on how the SOC affects nurses’ quality of life and job satisfaction. Ando and Kawano [28] examined the relationships among moral distress, SOC, mental health, and job satisfaction among 130 Japanese psychiatric nurses. They found that moral distress was negatively related to the SOC and job satisfaction. In Singapore, a study among hospital nurses showed that social support and the SOC predicted a high quality of life in all domains [29].

GRRs refer to “phenomena that provide one with sets of life experiences characterised by consistency, participation in shaping outcomes and an underload-overload balance” [19], p. 19. According to Antonovsky [19,30], such resources may include the following factors: (1) material resources (e.g., money), (2) knowledge and intelligence (e.g., knowing the real world and acquiring skills), (3) ego identity (e.g., integrated but flexible self), (4) coping strategies, (5) social support, (6) commitment and cohesion with one’s cultural roots, (7) cultural stability, (8) ritualistic activities, (9) religion and philosophy (e.g., stable set of answers to life’s perplexities), (10) preventive health orientation, (11) genetic and constitutional GRRs, and (12) individuals’ state of mind (see [31] for review). GRRs are factors that make it easier for people to perceive their lives as consistent, structured, and understandable, and thus prevent tension from being transformed into stress. A GRR is a generality, and an SRR is a particularity or context-bounded [21]. 

Research on salutogenesis has largely concentrated on the use of the two original questionnaires for measuring the SOC: the 29-item Orientation to Life Questionnaire and the 13-item questionnaire. The research is extensive and convincing. A strong SOC is related to positive individual health development but also health promotion at the group level, organisational level in healthcare settings, and among nurses. For further exploration of research on the function of the SOC and the core concepts of GRRs and SRRs, see *The Handbook of Salutogenesis* [20].

There are modified instruments, based on the salutogenic theory of health, adjusted for workplaces (WorkSoc) [32] for measuring work-related salutogenic factors: the Salutogenic Health Indicator Scale (SHIS) [33] and the Work Experience Measurement Scale (WEMS) [34]. More than 15 years have passed since Bringsén et al. [33] collected data among hospital staff (doctors, nurses, assistant nurses, rehabilitation staff, administrators, service personnel) and introduced the Salutogenic Health Indicator Scale (SHIS). At the same time, hospital employers (pre/post-op for ambulatory surgery and internal medicine wards) participated in focus-group interviews about their work experiences [34], which ended up in a new questionnaire, the Work Experience Measurement Scale (WEMS), particularly aiming at identifying work-related specific enhancing resources (SER). This was an attempt to broaden the discussion about specific resistance resources [19] to enhancing factors and health promotion. Common to these alternative instruments is that they measure salutogenic factors with their own questions and different response options to Antonovsky’s SOC scales. As far as we know, there are no other instruments developed based on the salutogenic theory and specifically focused on resistance resources in the context of hospital nurses.

## 2. Methods

### 2.1. Research Design

The study design is explorative. It is based on the salutogenic theory and its principles [19], i.e., it focuses on nurses’ resources and capacities to manage daily stressors in an increasingly demanding work environment. The item generation process was inspired by the MEASURE approach to instrument development by Kalkbrenner [35], Boateng et al. [36], and DeVellis [37], with the following steps. (1) **M**ake the purpose and rationale clear, which means to define the purpose of conducting an instrument development study by clarifying what it is seeking to measure and why the development of a new instrument is necessary, including a literature review of the construct and a summary of their review. Further, cite instruments that already exist and highlight a gap in the existing measurement literature [37]. (2) **E**stablish an empirical framework, which means identifying a theory from the literature review to set an empirical framework for the item development process. (3) **A**rticulate a theoretical blueprint, to decide which areas and concepts are to be investigated. (4) **S**ynthesising the content and scale development already done. (5) **U**se experts in the field for discussions of relevant research in the field. (6) **R**ecruit participants and administer the posting of the instrument. (7) **E**valuate validity and reliability via pilot testing.

### 2.2. Participants

The inclusion criteria for participating in the study were: (1) registered nurses (assistant, general, and specialist nurses); (2) working in close patient care at a hospital group in western Sweden; (3) working ≥ 50 percent to fulltime; and (4) understanding and speaking Swedish. The hospital group offers specialised care in many areas, such as emergency medicine, specialist medicine, surgical care, and adult psychiatric inpatient care to about 290,000 residents in the immediate area. Excluded were registered nurses who were (1) on sick, parental or study leave; (2) working < 50% of fulltime; (3) not working in close patient care; and (4) not understanding and speaking Swedish.

### 2.3. Data Collection

Data collection took place during January and February 2020, just before the first case of COVID-19 was found in the country. The convenience sample method was used for recruiting all available nurses in the hospital group that met the inclusion criteria. An electronic version of the questionnaire was administered online using the web-based Evaluation and Survey System (EvaSys), time-limited to the data collection period and only accessible to the invited participants. Three reminders were sent to the invited participants. Written informed consent was obtained before the respondents started to fill out the questionnaire. Participation was guided by the ethical principles of voluntary enrolment, privacy, and confidentiality. Participants could withdraw at any time without explanation. They were informed that the data would be treated according to the EU General Data Protection Regulation (GDPR). Ethical approval was obtained from The Swedish Ethical Review Authority (Dnr 2019-05185).

### 2.4. Data Analysis

Confirmatory factor analysis (CFA) presents a rigorous method for evaluating the measurement model, crucial for verifying a hypothesised structure. In this study, CFA was employed to authenticate whether the items accurately represent the proposed subscales (factors). This approach is grounded in the prior thematic analysis and theoretical underpinnings that shaped the factors and indicators. The quality assessment of the factor structure through the Mplus software encompasses three principal phases following the guidelines established by O’Rourke and Hatcher [38] and Byrne [39].

Phase 1: Evaluation of the Chi-Square Test. The initial phase involves applying the chi-square test to assess model–data congruence. A lower chi-square value coupled with a *p*-value approaching 1.00 typically signifies a favourable fit. However, in large samples or with real-world data, the chi-square statistic may indicate significance, suggesting potential model fit even when the model is appropriately specified. Hence, reliance on this test alone is inadequate, necessitating additional fit indices for a comprehensive evaluation.

Phase 2: Appraisal of Fit Indices. The second phase encompasses examining various global fit indices, including the comparative fit index (CFI), where values above 0.90 are indicative of an acceptable fit. the root mean square error of approximation (RMSEA) was also employed to gauge fitness by quantifying discrepancies between the implied and observed covariance matrices.

In this study, the evaluation of the fitness for all models was conducted utilizing the weighted least squares mean and variance-adjusted (WLSMV) chi-square statistic, along with several fit indices: the comparative fit index (CFI) and the root mean square error of approximation (RMSEA) including its 90% confidence interval, as outlined by Hu and Bentler [40] and Yu [41]. For the CFI, values exceeding 0.900 and 0.950 are indicative of an adequate and excellent fit, respectively. Similarly, RMSEA values below 0.080 and 0.060 correspond to an adequate and excellent fit, respectively.

Phase 3: Examination of Factor Loadings. The final phase involves scrutinizing the significance of the factor loadings, which delineate the magnitude of association between latent factors and their indicators. Insignificant factor loadings suggest that certain indicators may not be adequately capturing the essence of the latent factor, warranting their re-evaluation or elimination. For robust factor structures, factor loadings should ideally be 0.60 or higher, with those below 0.5 generally excluded from subsequent analyses. This critical step aids in affirming the validity of the factor structure.

In this study, the estimation of the measurement models was conducted utilizing Mplus 8.9 software, applying the robust weighted least squares (WLSMV) estimator. This method, as delineated by Muthén and Muthén [42], is superior in performance to the maximum likelihood estimator, particularly for ordered–categorical indicators with five or fewer response categories, as evidenced in the studies by Bandalos [43] and Li [44]. Additionally, the study estimated composite reliability (CR), with a threshold of ≥0.70 deemed acceptable, to gauge the internal consistency of each construct. This measure reflects the extent to which the individual indicators coherently represent their shared latent construct. The reliability assessment was further augmented by calculating the average variance extracted (AVE), where a value of ≥0.50 is considered satisfactory, indicating a substantial level of variance captured by the construct relative to the variance due to measurement error [45].

## 3. Results

### 3.1. Characteristics of the Sample

The study was carried out in a healthcare hospital group in western Sweden. A total of 782 nurses who met the inclusion criteria were invited to participate, of whom 475 completed the SalWork-N questionnaire. The response rate was 60.7%. The socio-demographic characteristics of the participants are presented (Table 1).

### 3.2. The Instrument Development Process

This study is part of a larger study, where data focused on factors that enhance nurses to manage work-related stress were collected. The instrument development process was not previously described and/or published.

### 3.3. The MEASURE Approach

The instrument development process was inspired by the MEASURE approach to instrument development by Kalkbrenner [35], Boateng et al. [36], and DeVellis [37]. *Making the purpose and rationale clear* is the first step. The purpose was to identify generalised and specific resistance resources among hospital nurses in close patient care to learn about their ability to manage their workload and the resources available. Existing research on a resource-oriented approach and instruments already developed was reviewed [20]. Two instruments were identified, with neither particularly directed to hospital nurses in close patient care and not specifically aimed at exploring GRRs and SRRs. In addition, interviews with nurses in close patient care with questions derived from the salutogenic theory and its core concept sense of coherence (SOC) were carried out and analysed via qualitative content analysis of the data by an inductive (data-driven) approach and a deductive approach for theoretically discussing the findings. Based on the theoretical idea and empirical data of salutogenesis, all items were created de novo. Seven areas (subthemes) emerged from the above-mentioned qualitative study as most relevant to the purpose of this study: job satisfaction, professional attitudes, workload, work motivation, commitment, belonging in working life, factors and conditions for remaining in the profession. To sum up, there is a knowledge gap in the existing measurement literature on generalised and specific resistance resources among hospital nurses. 

The next step in the process was to *establish empirical framework*. The established framework was the salutogenic theory by Antonovsky [19], a well-established theory of how people can manage stress and still maintain health. The theory is nowadays implemented not only at an individual level (here nurses), but at group (nursing team) and organisation (healthcare sector) levels [20].

The *articulated theoretical blueprint* was implemented to focus on the generalised and specific resistance resources against stress based on the salutogenic theory. A pool of 107 items with response alternatives was based on a Likert scale ranging from 1 (Not true at all) to 5 (Is very accurate), with the additional option of no point of view at all. Examples of questions are as follows: job satisfaction—“*I experience job satisfaction in my current work situation,”* or “*I feel that I can manage the workload at my job right now*”. (response alternatives: 1/Very low (Not true at all) to 5/Very high (Is very accurate)); contacts with patients—“*Patient contacts motivate me when I start a work shift,*” or “*Patient contacts motivate me to remain a nurse*” (1/To a very low degree (Not true at all) to 5/To a very high degree (Is very accurate)). The scale is a sum scale, with high scores corresponding to a high level of conformity.

The next step in the process was to *synthesise the content* and scale development already done. We found the Salutogenic Health Indicator Scale (SHIS) [33] and the Work Experience Measurement Scale (WEMS) [34].

*Experts in the field* of salutogenesis were *used* for discussions of relevant research. All researchers in the group were familiar with the salutogenic theory and how to construct surveys, as well as the design and methods used for the analysis. To reach consensus on which question areas were relevant for the new questionnaire, several workshops, and discussions within the research group were carried out. Discussions with experts in the field, among others, the inner core of researchers on salutogenesis, some of them former colleagues to Aaron Antonovsky, and a global network of salutogenesis, were carried out.

*Recruited participants* were registered hospital nurses in close patient care, who were invited to participate in the study.

*Evaluating the validity and reliability* was achieved by pilot testing as the final step in this process. A pilot test was conducted on nurses who were studying to become specialist nurses by giving them 30 min to answer the questionnaire. The students were informed about the aim of the study and its potential usefulness for nursing practice by one of the authors (HN). Informed consent was obtained before they started answering the questionnaire, and their anonymity was ensured. After the participants filled in the survey, one of the researchers (HN) carried out a follow-up by asking questions if any item was particularly unclear, if any question was difficult to respond to, or if the answer options were unclear. After all pilot tests, the answers received were discussed with the rest of the research group. Nine questionnaires were returned. A close examination of the answers indicated the need only for minor linguistic adjustments. For example, in the questions about forms of employment, some questions concerning commitment and belonging to the workplace were adjusted. In addition to background factors such as age, gender, professional education, length of experience as a nurse, and type of employment, the final SalWork-N questionnaire consisted of ten areas of interest. Subsequently, several changes and clarifications were made for both the questions that had been unclear or difficult to respond to, and some clarifications of the answer options to improve the survey. The pilot test was conducted in three separate groups on three different occasions, with 15–23 nurses participating in each group. After this review, all questionnaires were destroyed because they would not be used for further purposes. All nurses who participated in these pilot tests were informed before they filled in the questionnaire about how their answers and viewpoints would be taken care of and what would be done with the response forms.

### 3.4. Confirmatory Factor Analysis

This study employed confirmatory factor analysis (CFA) to explore the structure of workplace dynamics within a nursing context, focusing on 60 items across seven key domains: job satisfaction, professional role, work motivation, commitment, belonging in the workplace, factors and conditions for remaining in the profession, and workload. Each domain comprised various items and their standardised loadings were assessed to determine their significance in representing the latent constructs.

In the analysis, the overall model exhibited suboptimal fit indices (e.g., comparative fit index [CFI] = 0.677; root mean square error of approximation [RMSEA] = 0.197). Notably, the correlation between the latent variables of job satisfaction and work motivation exceeded the theoretical maximum of 1.00. This aberration suggests the potential existence of a Heywood case, a statistical anomaly often indicative of model misspecification or other underlying issues in the data or analysis. Furthermore, several indicators associated with the latent variables displayed standardised loadings below the threshold of 0.50, further questioning the adequacy of the model in capturing the underlying constructs effectively. This combination of factors—poor overall model fit, atypical correlation values, and low indicator loadings—necessitates a careful re-evaluation of the model’s structure and the operationalisation of its constructs. After removing indicators with standardised loadings lower than 0.60, a final measurement model emerged.

The overall fitness of the final model consisting of 21 items across seven domains was good. The CFI was found to be 0.956, indicating a good fit between the hypothesised model and the observed data. The RMSEA was 0.065, with a confidence interval of 0.059 to 0.072, suggesting a reasonable error of approximation. The chi-square test yielded a value of 508.752 with 168 degrees of freedom (*p* < 0.001), signifying a significant model fitness.

The standardised loadings for each domain range from moderate to high, indicating that most items are good indicators of their respective latent factors (shown in Table 2 below). Generally, loadings above 0.7 are considered very good, but even those above 0.6 can be acceptable in social science research.

The model demonstrates a strong internal consistency and convergent validity across all domains, as evidenced by the high values of composite reliability (≥0.70) and average variance extracted (≥0.50). These results indicate that the items within each domain are not only consistent with each other, but also effectively represent their respective latent constructs. However, the results from the confirmatory factor analysis (CFA) for this second-order model indicated a less than optimal fit. The comparative fit index (CFI), a key indicator of model fit, was found to be below the threshold of 0.95, a value generally considered indicative of a good model fit. This deviation from the acceptable standard suggests that the second-order model does not adequately capture the complexities and interrelationships of the seven domains within the context of our study.

The poor fitness of the second-order model underscores the complexity and multidimensionality inherent in the constructs representing workplace dynamics in healthcare. It suggests that the domains, while related, may not converge sufficiently to form a single overarching construct. This finding has implications for theoretical modelling and practical applications in organisational studies, particularly in healthcare settings, where the diversity of experiences and perceptions across different dimensions of job roles and environments is pronounced.

However, these results underscore the multidimensionality of workplace dynamics in nursing, reflecting the complex interplay of factors that influence job satisfaction, professional role, motivation, commitment, belonging, conditions for remaining in the profession, and workload management. The correlation patterns between the latent variables are illustrated in Table 3.

Notably, job satisfaction is closely linked to commitment and belonging in the workplace, suggesting that these areas are deeply interconnected in influencing employee satisfaction. Similarly, professional role correlates strongly with work motivation and workload, highlighting the significance of role definition and perceived responsibilities in shaping a healthcare professional’s motivation and perception of workload.

In addition, the square roots of the AVEs for each construct, shown in the diagonal, are all greater than the correlations involving their respective construct, thus satisfying the criteria for discriminant validity. This indicates that each construct is distinct and captures unique aspects of workplace dynamics.

In conclusion, while the first-order model successfully captured the intricacies of each domain, the second-order model failed to provide an adequate representation of the underlying structure of workplace dynamics. This emphasises the importance of considering the unique and individual contributions of each domain to the overall construct of workplace dynamics in healthcare settings. However, this refined measurement model, following the pruning of certain items through the CFA, now necessitates validation through empirical testing on an independent dataset. This step is crucial to ascertain the robustness and generalizability of the model in different data contexts.

## 4. Discussion

This study was conducted to describe the development of a new profession-specific questionnaire for measuring work-related resistance resources against stress among hospital nurses, followed by a psychometric test of the questionnaire. The integration of the seven domains of nursing experience with the core concepts of the sense of coherence (SOC), generalised resistance resources (GRRs), and specific resistance resources (SRRs) provides a comprehensive framework for understanding and improving the nursing profession. 

The results of the first-order confirmatory factor analysis (CFA) across seven domains offer insightful narratives when viewed through the lens of the salutogenic theory, particularly concerning generalised and specific resistance resources (GRRs and SRRs). Anchored in Antonovsky’s salutogenic model [19,20], these findings provide a deeper understanding of the nursing profession and the key factors contributing to a sustainable working life. For example, nurses play an indispensable role in the healthcare sector, and their well-being is critical. The capacity of nurses to maintain their health and avoid exiting the profession due to burnout, depression, or other work-related stressors is of utmost importance. Considering the demanding nature of nursing, marked by significant workloads, depression, burnout, and challenges amplified by pandemics [4,5,6], alongside the acute shortage of nurses in Europe [7,8], there is an urgent need to prioritise resources that bolster nurses’ resistance resources against stress. This approach extends beyond merely averting adverse outcomes like burnout [13,16] and depression [17]. It involves actively identifying and reinforcing resistance resources to counteract stress [19,20], promoting health [21], and understanding the various “push and pull factors” [11] in the workplace. Additionally, it includes exploring elements that foster professional fulfilment and growth [22,23]. Adopting this resource-oriented perspective, in line with the salutogenic model [19,20], underscores the importance of nurturing factors that encourage nurses to remain committed to their profession and workplace, thereby ensuring a robust and resilient healthcare system.

The Job Satisfaction domain’s high internal consistency underscores its role as a GRR. In the salutogenic model, job satisfaction is pivotal in contributing to a nurse’s sense of coherence, particularly regarding meaningfulness. This aligns with Eurofound’s [1] definition of sustainable work and supports the notion that satisfying work experiences enhance a nurse’s capacity to cope with stress, thereby promoting workplace health as defined by Vaandrager and Koelen [2].

In the professional role domain, the clarity and understanding of responsibilities mirror the characteristics of SRRs. This domain’s alignment with specific tools and knowledge essential for nursing echoes the salutogenic emphasis on resources that enhance manageability and comprehensibility [19,20], thus supporting nurses’ abilities to navigate their professional environment effectively.

Work motivation’s strong correlation with meaningfulness in the SOC context reflects the findings of Antonovsky [19] and subsequent research [24,25,26]. High motivation levels, as indicated in the CFA, suggest a reinforcing loop where motivated nurses find greater purpose in their work, enhancing their overall sense of coherence.

The commitment domain relates to the depth of a nurse’s engagement in their profession. The strong representation of this domain in the CFA results suggests that committed nurses are likely to possess a robust SOC, enabling them to manage workplace stress better. This is in line with research that associates a strong SOC with healthy functioning and job satisfaction [27,28,29].

Belonging in the workplace is identified as a key GRR, resonating with both GRRs and SRRs. A sense of belonging significantly contributes to manageability and comprehensibility, essential components of the SOC, reinforcing the notion that a supportive work environment is crucial for nurses’ well-being.

The factors and conditions for remaining domain highlights important aspects influencing nurses’ decisions to remain in their profession. These factors, identified as GRRs in the CFA results, contribute to a sustainable and health-promoting work environment, which is critical given the current shortage of nurses and the trend of nurses leaving the profession [7,8,9,10,11,12,13,14,15].

Finally, the workload domain’s association with manageability within the SOC framework underlines the importance of effective workload management. This is crucial for preventing burnout and ensuring nurse well-being, aligning with the necessity to maintain health and prevent departures from the profession, as highlighted in the introduction [4,5,6].

To sum up, the interplay between these seven domains with GRRs, SRRs, and the SOC constructs a holistic picture of the nursing work environment. Each domain contributes uniquely to enhancing nurses’ SOC, whether through fostering job satisfaction, clear professional roles, motivation, commitment, a sense of belonging, sustainable working conditions, or manageable workloads. This integrated approach underscores the importance of nurturing a supportive and resourceful environment for nurses, aligning with the salutogenic model’s emphasis on promoting health in the workplace.

### 4.1. Strengths and Limitations 

This study has several strengths and limitations. First, the new salutogenic questionnaire presented here, SalWork-N, is one of the next-generation questionnaires for measuring resistance resources related to nurses’ work situation. The results of this study are based on nurses’ own narratives of what works. The questions are profession-specific in that they are aimed at professional nurses in close patient care and emerged from face-to-face interviews with nurses in the same context. Second, the scale is applicable to a healthcare setting, particularly for nursing professionals with a focus on workplace health, which may contribute to a sustainable working life. It also addresses important factors that may make nurses decide to remain in the profession. In addition, nurses’ education may benefit from this new knowledge for their future professional life. 

The data collection was carried out shortly before the outbreak of the COVID pandemic. This can be seen as a limitation, as extensive research shows how the pandemic has increased the workload on nurses and their intention to leave. Thus, it becomes even more urgent to continue research on nurses’ resistance and enhancing resources to manage workload. On the other hand, the challenges brought about by the pandemic have perhaps particularly highlighted nurses’ life orientation and their capacity to handle stress, as well as the importance of the professional role. The awareness of these resources can strengthen the individual nurse, but also the healthcare leadership in their work to develop sustainable strategies to promote health among nurses. The key issue for the organisation is to *systematically* integrate the salutogenic perspective in all entities, policydocuments, and education and training for nurses, raising awareness of this way of working that looks at strengths and capacities without neglecting weaknesses. For the organisation, it is about developing sustainable work processes with clear structures, which make them comprehensible and meaningful. 

This is the first psychometric evaluation of the SalWork-N instrument. Thus, the use of confirmatory factor analysis (CFA) in this study represents a significant strength in our research methodology. The good model fitness obtained via CFA validates the existence and relevance of the seven domains identified in the thematic analysis. This statistical approach ensures that the structure of our model is not only theoretically sound but also empirically supported. By successfully applying CFA, we have established a robust framework for understanding the complexities within our thematic analysis, providing a reliable foundation for the further exploration and interpretation of the data. The model’s good fitness, as evidenced by indices such as the comparative fit index (CFI) and the root mean square error of approximation (RMSEA), underscores the effectiveness of our analytical methods and the potential applicability of these seven domains in understanding the underlying constructs of our study.

Despite these strengths, there are inherent limitations in our study. A primary limitation is the need to validate the seven domains identified through thematic analysis on a new dataset. The current study’s findings, while promising, are confined to the dataset used for this analysis. To establish the generalizability and replicability of these domains, further validation is required using an independent dataset. This step is crucial for confirming that the domains are not just specific to our current sample, but also relevant and applicable to other settings or populations. The validation process on a new dataset will help in refining the domains, potentially leading to adjustments in their definitions or structures based on new insights. This additional validation is essential for strengthening the credibility and utility of the identified domains in broader contexts. This is a matter for further research in the project.

### 4.2. Implications for Nursing Practice and Policy

The alignment of these seven domains based on the salutogenic theory underscores the importance of both GRRs and SRRs in nursing practice. To foster a resistant nursing workforce, it is vital to develop workplace policies and practices that enhance job satisfaction, clarify professional roles, motivate work, and ensure commitment and a sense of belonging. Additionally, understanding the factors that encourage nurses to remain in the profession can inform strategies to reduce turnover and promote sustainability in nursing careers.

The results of the study can be used in different therapies (family, cognitive) and in rehabilitation, where the focus is on patients’ abilities and capacities. Raising the question “when you have previously faced difficulties and felt stress, what helped you to deal with stress” starts a reflection that focuses on resources and opportunities that enhance nurses to overcome obstacles. Such a reflection requires training, to be aware of existing internal and external resistance resources. Further, a practical implication is to integrate the salutogenic approach in employee dialogues, structuring the dialogue in such a way that both perceive it comprehensible, manageable, and meaningful.

### 4.3. Future Research Directions and Implications

Further research can contribute by validating the instrument with more specific analysis on a considerably larger sample of nurses and health professionals. Being able to measure nurses’ work environment from a salutogenic perspective becomes useful for both the hospital leaders at different levels, as well as nurses in close patient care with an overall goal to create sustainable work environments. Nurses who enjoy their work are motivated to remain in the workplace as well as the profession; they also have good conditions for providing good patient care regardless of the context in which the care is provided. It is particularly important to identify the specific resistance resources against stress, as they are context-bounded and research on them is sparse.

## 5. Conclusions

The SalWork-N questionnaire is profession-specific in its character, adjusted for hospital nurses in close patient care and identifying resistance resources against stress. The results of the confirmatory factor analysis support a first-order seven-domain model of 21 items. The ability to manage workload was the most important component for nurses’ work environment, followed by working closely with patients. Nurses’ attitudes to work as a nurse and support from colleagues were additional important components for nurses to manage work-related stress.

## Figures and Tables

**Table 1:** Socio-demographic characteristics of the study population (N = 475) of nurses working in close patient care in a healthcare group in western Sweden.

Characteristics			N	%	
Gender					
Male			72	15.1	
Female			396	83.4	
No response			7	1.5	
Civil status					
In a relationship			383	80.7	
Not in a relationship			88	18.5	
No response			4	0.8	
Children					
Have child			209	44.0	
No child			256	53.9	
No response			10	2.1	
Education					
Nurse			329	69.3	
Specialist nurse			104	21.9	
Both			36	7.6	
No response			6	1.2	
Academic examination					
Candidate			257	54.1	
Master			71	15.0	
Licentiate			2	0.4	
No response			145	30.5	
Time as a nurse					
<1 year			19	4.0	
1–2 years			40	8.4	
3–5 years			81	17.1	
6–10 years			70	14.7	
11–20 years			115	24.3	
20–30 years			78	16.4	
>30 years			68	14.3	
No response			4	0.8	
Workplace					
County healthcare			459	96.7	
Private care provider			1	0.2	
Other			3	0.6	
More than one response			10	2.1	
No response			2	0.4	
Percent of work					
100%			468	98.6	
50%			4	0.8	
Other			1	0.2	
No response			2	0.4	
Type of employment					
Part-time			24	5.1	
Hourly			439	92.4	
More than one response			1	0.2	
No response			11	2.3	
Shift type					
Day			250	52.6	
Night			52	11.0	
Rotation in shift			165	34.7	
More than one response			6	1.3	
No response			2	0.4	
	N	%	Mean	SD	Range
Age			43.0	12.5	23–72
23–30 years	102	21.5			
31–40 years	112	23.6			
41–50 years	95	20.0			
51–60 years	110	23.2			
61–69 years	39	8.2			
>70 years	3	0.6			
No response	14	2.9			

**Table 2:** Final measurement model of the 21-item Salutogenic Survey on Sustainable Working Life for Nurses (SalWork-N) scale among Swedish nurses working in close patient care in a healthcare group (N = 475) across seven domains. Standardised coefficients, composite reliability, and average variance extracted for the latent variables.

Domains	Standard- Ised Loadings	S.E.	*p*-Value	C.R.	AVE
**Job Satisfaction**				0.824	0.609
Importance of job satisfaction	0.834	0.058	<0.001		
Importance of humor and camaraderie at work	0.751	0.050	<0.001		
Contribution of colleagues to job satisfaction	0.754	0.045	<0.001		
**Professional Role**				0.773	0.534
Creativity in the nursing role	0.664	0.030	<0.001		
Satisfaction with the current nursing job	0.820	0.030	<0.001		
Pride in being a nurse	0.698	0.032	<0.001		
**Work Motivation**				0.834	0.626
Importance of patient contact	0.799	0.030	<0.001		
Influence of patients’ relatives	0.772	0.029	<0.001		
Responsibilities in nursing	0.803	0.031	<0.001		
**Commitment**				0.826	0.707
Engagement in current job	0.928	0.039	<0.001		
Importance of having engaged colleagues	0.743	0.038	<0.001		
**Belonging in the Workplace**				0.888	0.799
Sense of belonging to a workgroup	0.941	0.031	<0.001		
Sense of belonging among nursing colleagues	0.844	0.028	<0.001		
**Factors and Conditions for Remaining** **in the Profession**				0.900	0.750
Importance of leisure time	0.828	0.023	<0.001		
Family influence on work	0.898	0.023	<0.001		
Friends’ influence on work	0.870	0.019	<0.001		
**Workload**				0.880	0.596
Handling current workload	0.823	0.022	<0.001		
Personal resources for handling work challenges	0.772	0.024	<0.001		
Work-related resources for handling challenges	0.800	0.022	<0.001		
Organisational resources for handling challenges	0.694	0.030	<0.001		
Work–life balance	0.764	0.025	<0.001		
CFI = 0.956					
RMSEA = 0.065 (0.059; 0.072)					
Chi-Square Test of Model Fit = 508.752, (df) 168, *p* ≤ 0.001					

Note: S.E. = standard error, df = degrees of freedom, AVE = average variance extracted, C.R. = composite reliability, CFI = comparative fit index, RMSEA = root mean square error of approximation with 90% confidence intervals within parenthesis.

**Table 3:** Correlations between the latent variables.

Latent Variables	1	2	3	4	5	6	7
1. Job Satisfaction	(0.781)						
2. Professional Role	**0.339**	(0.730)					
3. Work Motivation	**0.275**	**0.716**	(0.791)				
4. Commitment	**0.530**	**0.598**	**0.596**	(0.841)			
5. Belonging in the Workplace	**0.620**	**0.466**	**0.324**	**0.431**	(0.891)		
6. Factors and Conditions for Remaining in the Profession	**0.404**	**0.179**	**0.230**	**0.210**	**0.321**	(0.866)	
7. Workload	0.128	**0.646**	**0.384**	**0.298**	**0.329**	−0.035	(0.772)

**Note:** Values in parentheses in the diagonal represent the square root of the average variance extracted (AVE) for each construct, which is used to assess discriminant validity. Bold values indicate statistical significance.

## Data Availability

The data are unavailable due to privacy or ethical restrictions. The data that support the findings of this study are available from the corresponding author upon reasonable request.
